# The role of LPA and YAP signaling in long-term migration of human ovarian cancer cells

**DOI:** 10.1186/1478-811X-11-31

**Published:** 2013-04-24

**Authors:** Hui Cai, Yan Xu

**Affiliations:** 1 First Affiliated Hospital, Xi’an Jiaotong University, Xi’an, China; 2 Department of Obstetrics and Gynecology, Indiana University School of Medicine, 975 W. Walnut St. IB355A, Indianapolis, IN 46202, USA

**Keywords:** EOC, LPA, YAP, TAZ, Lats, PP1A, Amphiregulin (AREG)

## Abstract

**Background:**

The Hippo-YAP signaling pathway is altered and implicated as oncogenic in many human cancers. However, extracellular signals that regulate the mammalian Hippo pathway have remained elusive until very recently when it was shown that the Hippo pathway is regulated by G-protein-coupled receptor (GPCR) ligands including lysophosphatidic acid (LPA) and sphingosine 1-phosphophate (S1P). LPA inhibits Lats kinase activity in HEK293 cells, but the potential involvement of a protein phosphatase was not investigated. The extracellular regulators of YAP dephosphorylation (dpYAP) and nuclear translocation in epithelial ovarian cancer (EOC) are essentially unknown.

**Results:**

We showed here that LPA dose- and time-dependently induced dpYAP in human EOC cell lines OVCA433, OVCAR5, CAOV3, and Monty-1, accompanied by increased YAP nuclear translocation. YAP was involved in LPA-induced migration and invasion of EOC cells and LPA_3_ was a major LPA receptor mediating the migratory effect. We demonstrated that G_13_, but not or to a lesser extent G_12_, G_i_ or G_q_, was necessary for LPA-induced dpYAP and its nuclear translocation and that RhoA-ROCK, but not RhoB, RhoC, Rac1, cdc42, PI3K, ERK, or AKT, were required for the LPA-dpYAP effect. In contrast to results in HEK293 cells, LPA did not inhibit Mst and Lats kinase in OVCA433 EOC cells. Instead, protein phosphatase 1A (PP1A) acted down-stream of RhoA in LPA-induction of dpYAP. In addition, we identified that amphiregulin (AREG), a down-stream target of YAP which activated EGF receptors (EGFR), mediated an LPA-stimulated and EGFR-dependent long-term (16 hr) cell migration. This process was transcription- and translation-dependent and was distinct from a transcription- and YAP-independent short-term (4 hr) cell migration. EOC tissues had reduced pYAP levels compared to normal and benign ovarian tissues, implying the involvement of dpYAP in EOC pathogenesis, as well as its potential marker and/or target values.

**Conclusions:**

A novel LPA-LPA_3_-G_13_-RhoA-ROCK-PP1A-dpYAP-AREG-EGFR signaling pathway was linked to LPA-induced migration of EOC cells. Reduced pYAP levels were demonstrated in human EOC tumors as compared to both normal ovarian tissues and benign gynecologic masses. Our findings support that YAP is a potential marker and target for developing novel therapeutic strategies against EOC.

## Background

Many pathways originally identified for their function in development have subsequently been shown to be involved in tumorigenesis. Among them, the Hippo-YAP (Yes-associated protein) signaling pathway plays a key role in the regulation of organ size by inhibiting cell proliferation, promoting apoptosis, and limiting stem/progenitor cell expansion in epithelial tissues [1]. YAP was originally identified using antibodies against the amino terminal domain of the Yes protein [2] and is negatively regulated by Hippo-pathway kinases via phosphorylation of Ser127, which results in YAP 14–3–3 binding, cytoplasmic retention, and degradation [3]. Bioactive lipids LPA and sphingosine-1-phosphate (S1P) were recently identified as extracellular regulators of YAP signaling in HEK293 and mammary cell lines [1,4].

The Hippo-YAP pathway is altered and implicated as oncogenic in a variety of human cancers, including epithelial ovarian cancer (EOC). In particular, high levels of nuclear YAP (nYAP), or low levels of cytoplasmic phosphorylated YAP (cpYAP), are associated with poor survival from EOC. *In vitro* assays show that YAP is involved in increased cell proliferation, resistance to cisplatin-induced apoptosis, faster cell migration, and anchorage-independent growth in EOC OVCA432 and OVCAR8 cells [5,6]. However, the extracellular regulators and detailed mechanisms of YAP signaling in EOC cells are essentially unknown.

The oncogenic role of bioactive lipids, especially LPA, in EOC cells has been amply demonstrated by our lab and others; LPA promotes tumor cell proliferation, survival, adhesion, migration, invasion, and metastasis *in vitro* and *in vivo*[7-14]. LPA acts through six known G protein-coupled receptors (LPA_1-6_) [15,16]. We have shown that LPA_2_ and LPA_3_, but not LPA_1_ or LPA_4_, are involved in LPA-induced cell migration of EOC cells [7,9,10,17,18]. Both LPA_1_ and LPA_3_ were implicated in LPA-induced YAP activation in HEK293 cells [1]. LPA_3_ has been shown to be coupled predominately to G_q_ proteins and also to G_i_ proteins [19-21], but not previously to G_12_ and/or G_13_.

YAP is a transcriptional co-activator [1], but the downstream targets of YAP pertinent to cancer cell migration have been only minimally studied. Interestingly, amphiregulin (AREG), an epidermal growth factor receptor (EGFR) ligand, has been identified as a target of both YAP and TAZ; AREG is a secreted factor that contributes to YAP-mediated cell proliferation and migration in MCF10A and neighboring cells [22,23].

The importance of LPA signaling in EOC prompted our investigation of the potential regulation of YAP by LPA in EOC cells. YAP activation was assessed by its dephosphorylation (dp) and nuclear translocation. The effects of YAP activation on cell migration and invasion were studied. The YAP signaling pathway in EOC cells was identified using pharmacological reagents and genetic forms of signaling genes, as well as siRNAs. Importantly, YAP activation in human tumor, benign, and normal tissues was examined to demonstrate the translational potential of this pathway in EOC.

## Results

### LPA dose- and time-dependently induced dpYAP in EOC cells

**Figure 1:**
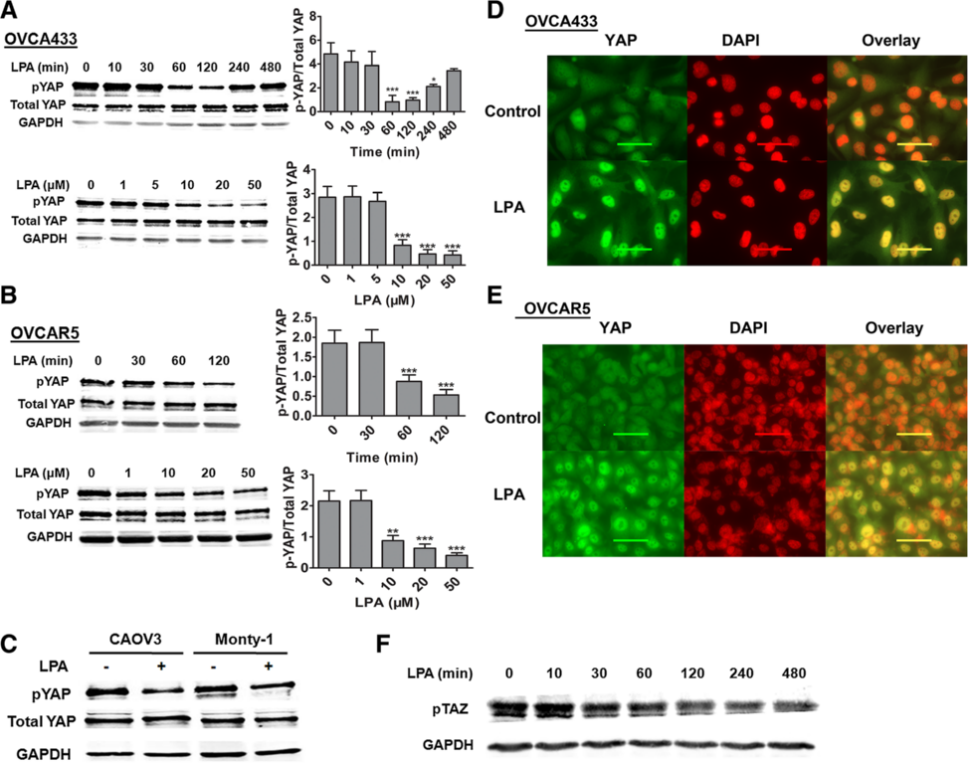
**LPA-induced dpYAP and YAP nuclear translocation in EOC cells.** OVCA433 (**A**) and OVCAR5 (**B**) cells were starved for 16 hr, then treated with LPA (10 μM) for different times or with different concentrations of LPA for 2 hr. Western blots analyses and quantification methods were described in Materials and Methods. Representative results are shown from three independent experiments. *P < 0.05, **P < 0.01, ***P < 0.001. **C**, LPA (10 μM, 2 hr)-induced dpYAP was tested in two more EOC cell lines. **D** and **E**, LPA-induced YAP nuclear translocation is shown in OVCA433 and OVCAR5 cells. Green: YAP; red: DAPI. **F**, LPA (10 μM) time-dependently induced de-phosphorylation of TAZ.

We tested whether LPA affected the dephosphorylation of YAP (dpYAP) at ser127 in EOC cells. LPA induced dpYAP in a dose- and time-dependent manner in OVCA433 cells with the maximal effect at 2 hr and at 20 μM of LPA (Figure 1A). Similar LPA effects on dpYAP were observed in a different EOC cell line OVCAR5 (Figure 1B). In addition, we tested two more EOC cell lines, CAOV3 and Monty-1, and found that LPA also induced dpYAP in these cells (Figure 1C). Concomitantly, LPA induced YAP nuclear translocation in both EOC cell lines tested (OVCA433 and OVCAR5) (Figure 1D and 1E). These results indicate that as in HEK293 cells [1], LPA is an extracellular regulator of YAP activation in EOC cells, and the effect is not limited to one EOC cell line. Since TAZ is a paralog of YAP, we also tested the effect of LPA on dephosphorylation of TAZ (dpTAZ). As shown in Figure 1F, LPA also induced dpTAZ in OVCA433 cells, although the time-dependence of the effect was different.

### LPA-induced cell migration and invasion was YAP-dependent

**Figure 2:**
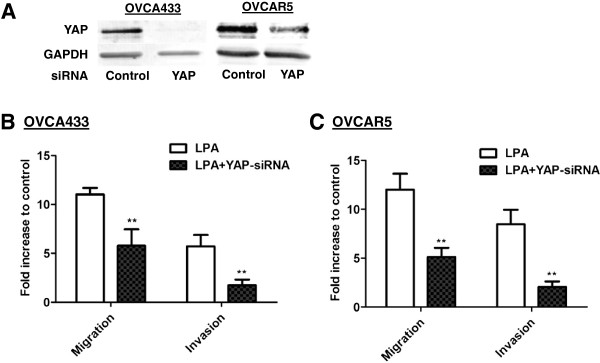
**LPA-induced migration and invasion of EOC cells was reduced by YAP-siRNA. A**, Reduced YAP expression by YAP siRNA in OVCA433 and OVCAR5 cells detected by Western blot. **B** and **C**, The effect of down-regulation of YAP on cell migration and invasion induced by LPA (10 μM) in OVCA433 and OVCAR5 cells (conducted 48 hr-post siRNA treatment). The results are from three independent experiments. **P < 0.01.

The potential involvement of YAP in LPA-induced cellular functions was tested. YAP was effectively down-regulated using siRNA (Figure 2A) and LPA-induced migration (tested at 16 hr) and invasion (tested at 24 hr) (Figure 2B and 2C) were significantly reduced in both OVCA433 and OVCAR5 cell lines, supporting the functional role of YAP in LPA signaling.

### LPA-induced dpYAP and nuclear translocation of YAP was RhoA**-**ROCK dependent

**Figure 3:**
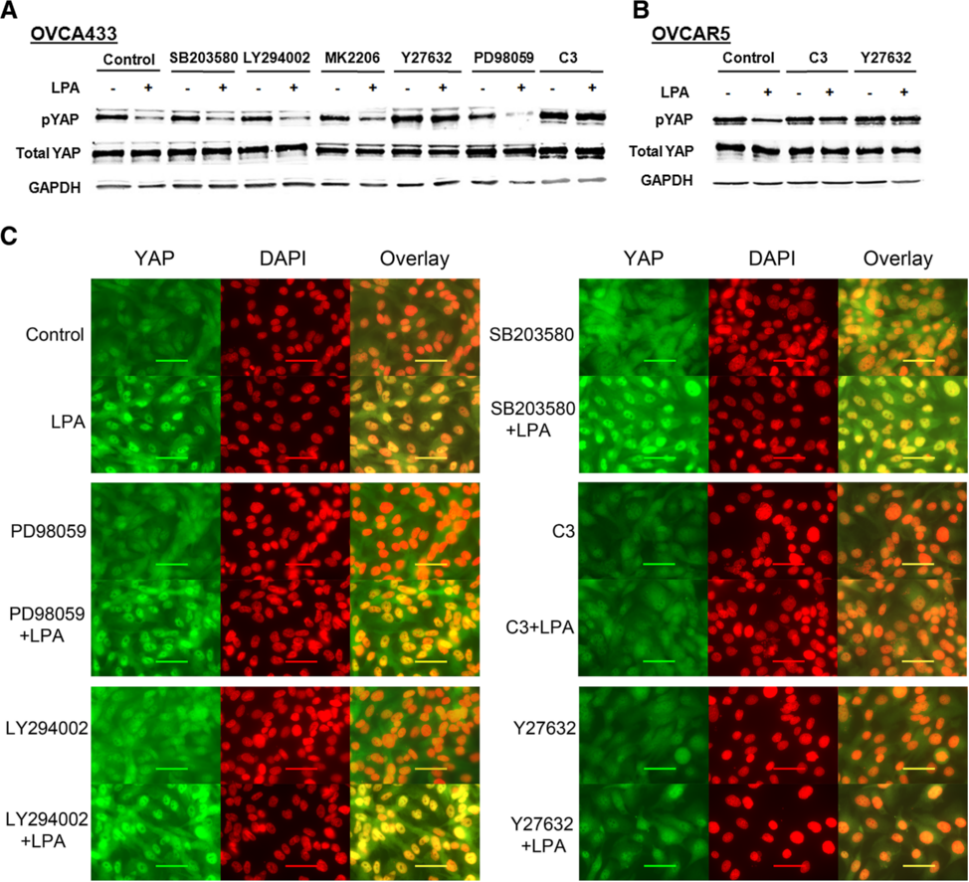
**LPA-induced dp-YAP and YAP nuclear translocation were dependent on Rho/ROCK, but independent of PI3K, MEK, p38, and AKT.** After starved from FBS for 16 hr, cells were pretreated with different inhibitors, SB203580 (10 μM), LY294002 (10 μM), Ki16425 (10 μM), MK2203 (1 μM), Y27632 (10 μM), PD98059 (30 μM) for 1 hr, and C3 (1 μg/mL) for 2 hr prior to stimulation with LPA (10 μM, 2 hr). pYAP expression in OVCA433 (**A**) and OVCAR5 (**B**) cells was analyzed by Western blot. **C**, OVCA433 cells were treated as described in **A**. Cells were fixed and stained for YAP (green). Cell nuclei were stained with DAPI (red). Representative results are shown.

LPA activates cellular effects via G_i_-PI3K-MAPK, G_q_-PLC-PKC, and/or G_12/13_-Rho pathways [19,24]. Selective pharmacological inhibitors and reagents were used to dissect the signaling pathway leading to the LPA-YAP effects. LPA (10 μM for 2 hr)-induced dpYAP and nuclear translocation of YAP were not affected by the PI3K-Akt or MAP kinase (p38; MEK-ERK) pathways, but were completely abolished by the Rho inhibitor C3 transferase, as well as by the Rho-kinase (ROCK) inhibitor Y27632 in OVCA433 cells (Figure 3A). C3 transferase and Y27632 also blocked LPA-induced dpYAP in OVCAR5 cells (Figure 3B). The pharmacological sensitivities of LPA-induced YAP nuclear translocation were consistent with the dpYAP revealed by Western blot analyses, suggesting that the two processes are closely coupled (Figure 3C).

### LPA_3_, G_13_, and RhoA-ROCK were involved in mediating LPA-induced dpYAP

**Figure 4:**
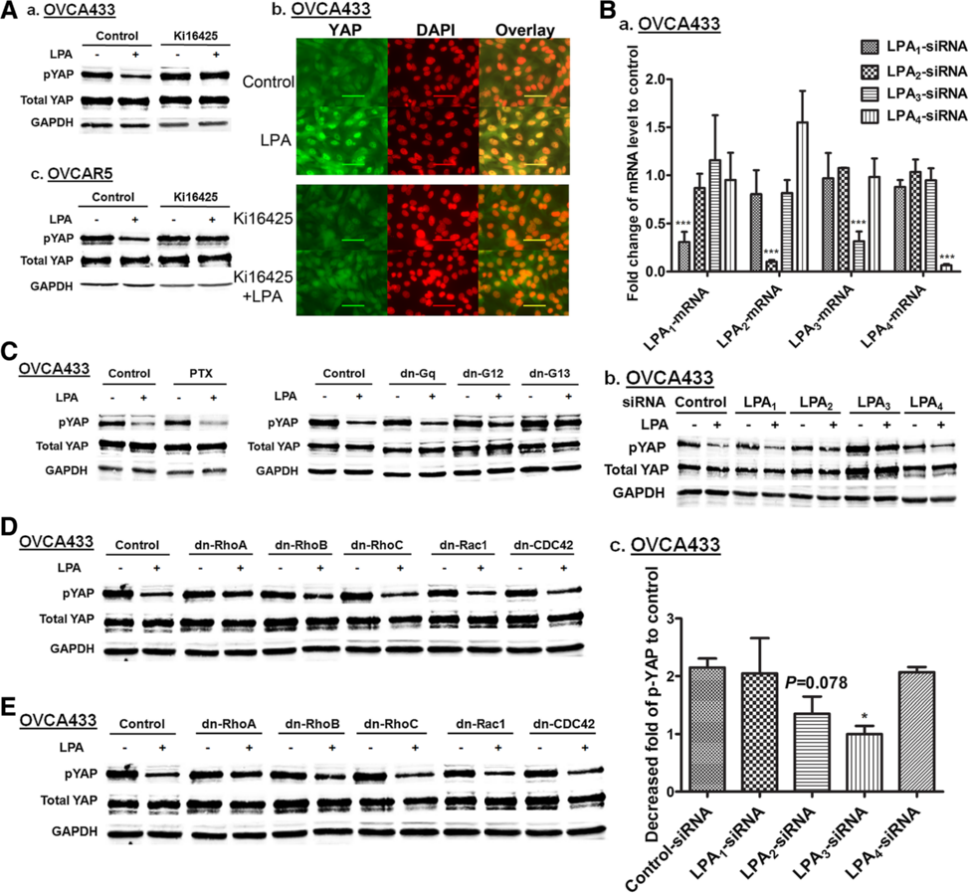
**LPA**_**3**_**, but not or to lesser extent LPA**_**1**_**, LPA**_**2**_**, and LPA**_**4**_**, mediated the LPA-dpYAP effect. A**, OVCA433 (**a**) and OVCAR5 (**c**) cells were starved and pretreated with Ki16425 (10 μM) for 1 hr prior to treatment with LPA (10 μM, 2 hr). pYAP was analyzed by Western blot. (**b**) The effect of Ki16425 on LPA (10 μM, 2 hr)-induced YAP nuclear translocation in OVCA433 cells. Green: YAP; red: DAPI. Representative results are shown. **B**, (**a**) The mRNA levels of LPA receptors after siRNA-treatment in OVCAR433 cells were determined by quantitative real-time PCR. Normalized expression values are given as percentage of control siRNA treated samples (means ± SD of three independent experiments). ***P < 0.001. (**b**) LPA (10 μM, 2 hr)-induced dpYAP effects were determined in LPA receptor specific siRNA-treated cells (48 hr post-transfection). (**c**) Quantitation of Western blots from (**b**) presented as fold decrease of pYAP after LPA stimulation compared to unstimulated controls. The data are means ± SD from three independent experiments. *P < 0.05. **C**, **D** and **E**, Cells were pretreated with PTX (100 ng/mL, 16 hr) or transfected with different dn plasmids for 48 hr, starved and then treated with LPA (10 μM, 2 hr). Cell lysates were analyzed by Western blot. Representative results are shown.

Most EOC cell lines express LPA_1-3_ receptors. Ki16425, a dual inhibitor for LPA_1_ and LPA_3_ inhibited LPA-induced dpYAP and nuclear translocation of YAP in OVCA433 cells (Figure 4A), suggesting that one or both of these receptors are involved. Selective blockage of LPA_1-4_ was achieved utilizing specific siRNAs as assessed by quantitative PCR (Figure 4B). Down-regulation of LPA_3_, but not LPA_1_ or LPA_4_, reversed LPA-induced dpYAP in OVCA433 cells (Figure 4B). Although down-regulation of LPA_2_ resulted in reduced dpYAP, three independent experiments showed that the effect was not statistically significant (*P* = 0.078) (Figure 4B, c). Additional studies in OVCA433 and other cell lines are needed to further define the role of LPA_2_ in LPA-YAP effect.

Pertussis toxin (PTX), a specific inhibitor of G_i_ protein, and dominant negative (dn)-forms of G proteins were used to determine which trimeric (large) and small G proteins were involved. LPA-induced dpYAP was insensitive to PTX (Figure 4C), suggesting that G_i_ proteins were not involved. The results from cells transfected with different dn-forms of large and small G proteins showed that G_13_ and RhoA were necessary for the LPA-induced dpYAP. The experiments indicated that G_q_, Rac1, cdc42, RhoB, and RhoC, were not at all or much less involved in the effect, and G_12_ may be involved to a small extent (Figure 4D and 4E).

### A protein phosphatase, PP1A, played an important role in the LPA-YAP effect in EOC cells

**Figure 5:**
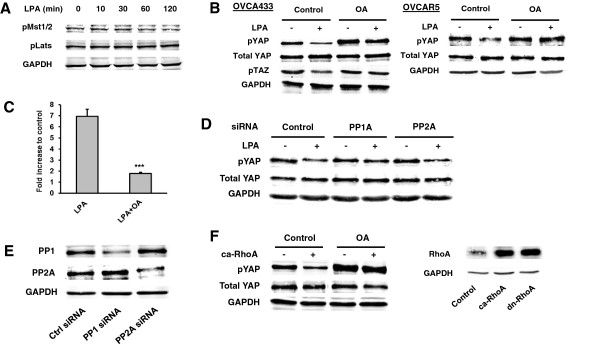
**PP1A was involved in LPA-induced dpYAP and cell migration. A**, Starved OVCA433 cells were treated with LPA (10 μM) for different times, and pMst1/2 and pLats were analyzed by Western blot. **B**, Starved OVCA433 and OVCAR5 cells were pretreated with OA (100 nM, 1 hr), followed by LPA (10 μM, 2 hr). pYAP and pTAZ were analyzed by Western blot. **C**, OVCA433 cells were treated as described in (**B**) and the effect of OA on cell migration was tested. *** *P* < 0.001. **D**, OVCA433 cells were transfected with control, PP1A, or PP2A siRNAs for 48 hr. The cells were starved for 16 hr and treated with or without LPA (10 μM, 2 hr). Cell lysates were analyzed by Western blot for pYAP. **E**, Specific down-regulation of PP1A or PP2A proteins. **F**, OVCA433 cells were transfected with the vector or the ca-RhoA plasmid, and then treated with or without OA (100 nM, 4 hr). RhoA expression was examined in cell lysates by Western blot analyses.

Yu *et al.* have shown that LPA induces dpYAP mainly via suppression of Lats1/2, but does not have effects on Mst [1]. We tested the effect of LPA on Mst and Lats in EOC cells. Consistent with the results in HEK293 or MEFs [1], LPA did not induce changes in pMst [Mst1 (T183) and Mst2 (T180)] (Figure 5A). However, in contrast to the results in HK293 cells, LPA (10 μM) did not affect pLats (S909) during the same time period when it induced dpYAP (0–2 hr) (Figure 5A).

LPA-induced dpYAP could be mediated by activation of its protein phosphatase (PP). Interestingly, the catalytic subunit of protein phosphatase-1 (PP1A) has been shown to dephosphorylate YAP to induce its nuclear accumulation and transcriptional activation in Hela and HEK293 cells, and is associated with resistance to cisplatin in YAP-transfected EOC cells [25]. Okadaic acid (OA; 100 nM), an inhibitor of PP1A and PP2A, almost completely reversed the LPA-dpYAP effect in both OVCA433 and OVCAR5 cells (Figure 5B), and strongly inhibited LPA-induced cell migration in OVCA433 cells (Figure 5C), suggesting that one or more protein phosphatases (PPs) are involved in dpYAP in EOC cells. OA treatment also reversed LPA-induced dpTAZ (Figure 5B), consistent with an important role for a PP in LPA-induced dephosphorylation of YAP and TAZ in OVCA433 cells.

To determine which PP was involved, siRNAs against the catalytic subunits of PP1 and PP2 were used. LPA-induced dpYAP was reversed by the PP1A but not the PP2A siRNA, suggesting that PP1A is activated by LPA and YAP is likely to be a direct substrate of PP1A (Figure 5D). The specificity of the siRNA down-regulation of PP1A and PP2A is shown in Figure 5E.

To determine whether PP1A is up- or down-stream of RhoA-ROCK, we used the constitutively active (ca) form of RhoA (G14V). The ca-RhoA was able to induce dpYAP in an OA-sensitive manner, suggesting that PP1A was down-stream of RhoA (Figure 5F). The expression of transfected RhoA was confirmed using RhoA antibody (Figure 5F).

### LPA-induced AREG secretion and EGFR-dependent cell migration was LPA_3_-G13-RhoA-ROCK-PP1-dpYAP-dependent

**Figure 6:**
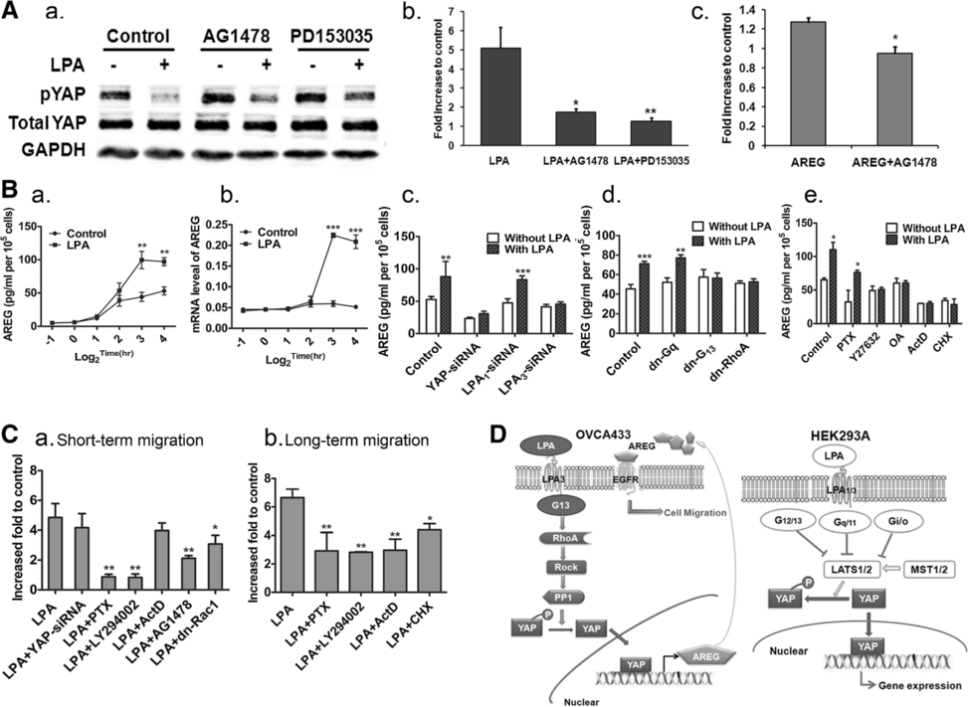
**YAP-dependent amphiregulin (AREG) production/secretion mediated EGFR-dependent LPA-induced cell migration. A**, The effects of AG1478 (1 μM, 1 hr pretreatment) and PD153035 (4 μM, 1 hr pretreatment) on LPA-dpYAP (**a**), or cell migration (**b**); AREG (100 pg/mL) induced AG1478 (1 μM, 1 hr)-sensitive migration of OVCA433 cells (**c**). *P < 0.05. **B**, Basal and LPA (10 μM)-induced AREG in conditioned media (CM) of OVCA433 cells (**a**). mRNA level of AREG was increased by LPA (**b**). LPA (10 μM, 8 hr)-induced AREG in CM was YAP- and LPA_3_-, but not LPA_1_-dependent (**c**). LPA-induced AREG production/secretion was blocked by dn-G_13_, dn-RhoA, but not PTX (100 ng/mL, 16 hr) or dn-G_q_ (**d** and **e**). **C**, LPA-induced short-term migration (4 hr) was not dependent on YAP or transcription (ActD, 1 μg/mL, 1 hr pretreatment), but was sensitive to PTX (100 ng/mL, 16 hr), LY294002 (10 μM, 1 hr pretreatment) and dn-Rac1 transfection (**a**). LPA-induced long-term cell migration was sensitive to ActD and CHX treatment (20 μg/mL, 1 hr pretreatment), as well as PTX and LY294002 (**b**). Data are from three independent experiments (means ± SD). *P < 0.05, **P < 0.01. **D**, Summary the LPA-YAP signaling pathway revealed in this work (OVCA433 cell line, left panel) and the previous work [1] (HEK293A cell line, right panel).

The mechanisms by which YAP signaling affects cell migration has been only minimally studied. Since YAP is a transcriptional co-activator [1] and AREG, an EGFR ligand, has been identified as a YAP and TAZ target [22,23], we tested whether EGFR was involved in LPA-induced cell migration in a YAP-dependent manner. We found that AG1478, an EGFR selective inhibitor, did not inhibit LPA-induced dpYAP, but did inhibit LPA-stimulated cell migration (Figure 6A, a, b), suggesting that an EGFR ligand may be a target of YAP. We tested AREG directly and showed that indeed, AREG induced an AG1478-sensitive cell migration (Figure 6A, c). The involvement of EGFR and AREG was further supported by the actions of a second EGFR inhibitor, PD153035 [26] on both pYAP and migration (Figure 6A, a and b).

OVCA433 cells demonstrated a basal secretion of AREG, as measured by an increased AREG in conditioned medium over time. LPA (10 μM) stimulated AREG secretion above the basal level, correlated to the increase in AREG mRNA expression, which peaked at 8 hr (Figure 6B, a, b). An siRNA against YAP reduced both basal and LPA-induced AREG secretion from the OVCA433 cells (Figure 6B, c). To confirm the signaling pathway, we tested the potential involvement of several key molecules in AREG section. As shown in Figure 6B, c, down-regulation of YAP and LPA_3_, but not LPA_1_, completely abolished LPA-induced AREG secretion. LPA-induced AREG secretion was also sensitive to dn-G_13_, dn-RhoA, Y27632, and OA, but not PTX or dn-G_q_ (all tested at 8 hr) (Figure 6B, d and e), consistent with the LPA-induced YAP signaling pathway. In addition, LPA-induced AREG secretion was actinomycin D (ActD) and cyclohexamide (CHX)-sensitive, suggesting that both transcription and translation processes are involved (Figure 6B, e).

LPA-induced cell migration has been extensively studied in EOC and other cancer cells [7,10,17,27,28]. However, the current work is the first to show the involvement of YAP in LPA actions in EOC cells. Relatively short times (~ 4 hr) are traditionally used for Transwell migration studies in EOC cells. Since YAP is a transcriptional co-activator, we tested migration over a longer time (all migration data shown above were from 16 hr assays). To compare the involvement of YAP in short- versus long-term migration, we conducted parallel studies and identified two phases of LPA-induced cell migration. The short-term LPA-induced cell migration was YAP and transcription-independent (Figure 6C, a), and highly sensitive to PTX and LY294002, suggesting that this process was mainly controlled by a G_i_-PI3K pathway. Similarly, Rac is well-known to be involved in LPA-induced cell migration [29,30] and we showed that dn-Rac 1 inhibited LPA-induced short-term migration (Figure 6C, a). In contrast, the long-term LPA-induced migration was YAP-, transcription-, and translational-dependent (Figures 6C, b and 2B, 2C). It was not surprising to find that the long-term migration was also sensitive to PTX and LY294002, but to a lesser extent (Figure 6C, b), since the long-term migration was comprised of both short- and long-term phases. Interestingly, both short- and long-term migration were partially AG1478-sensitive (Figure 6C, a and 6A, b), suggesting that at least two different mechanisms were involved in LPA-EGFR crosstalk in these cells.

### pYAP was significantly decreased in human EOC tissues vs. normal and benign tissues

**Figure 7:**
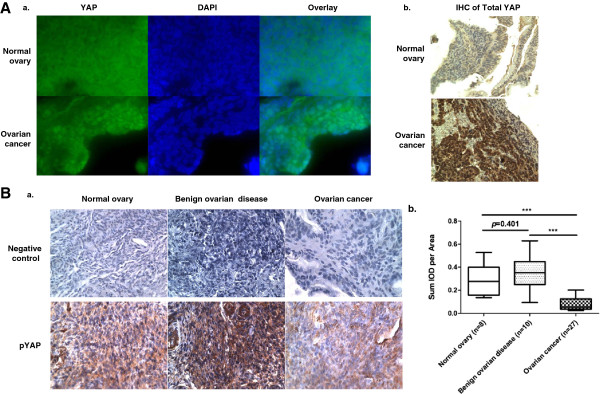
**pYAP expression was low in EOC tissues when compared to both normal and benign ovarian tissues. A**, Immunofluorescence staining of total YAP (green) in normal and EOC tissues showed that YAP was mainly cytosolic and nuclear (blue) in normal and EOC tissues, respectively (**a**). Different locations for total YAP in normal and EOC tissues were confirmed by IHC (**b**). **B**, EOC tissues express lower levels of pYAP than normal and benign tissues. The top row shows the negative controls of IHC when the first antibody was not added. Representative pictures are shown (**a**). Summary of the quantified IHC results from normal ovary (n = 8), benign ovarian tissues (n = 10), and EOC tissues (n = 27) (**b**).

We tested human tissues (normal, benign gynecological mass, and EOC tumor) for the presence and cellular location of total YAP and/or pYAP. The demographic and clinical data for the subjects are shown in Tables 1 and 2. As shown in Figure 7A, although both normal and EOC tissues express YAP, the protein was differentially located in the cytoplasm and the nucleus of normal and the EOC tissues, respectively. In addition, EOC tissues had lower levels of pYAP (brown color indicates positive staining; Figure 7B shows representative data). These results are highly consistent with two recent studies [5,6], but this is the first time that pYAP was examined and detected in benign tissues.

**Table 1:** Patient demographic data

	**n**	**Age (years)**		**Race (n)**		
		**Mean**	**SD**	**White**	**African American**	**Unknown**
Healthy	8	60.3	13.8	5	3	0
Benign disease	10	64.2	13.9	10	0	0
Ovarian Cancer	27	57.4	10.7	16	3	8

**Table 2:** Clinical characteristics for the ovarian cancer patients

	**Number**	**Percent**
Stage		
I	2	7.4
II	6	22.2
III	16	59.3
IV	3	11.1
Tumor type		
Serous	24	88.9
Endometriod	1	3.7
Stromal tumor	1	3.7
Unknown	1	3.7
Tumor grade		
I	0	0
II	3	11.1
III	24	88.9

## Discussion

Data shown here support the novel concept that LPA can stimulate two potentially overlapping phases of cell migration with short- and long-terms (4 and 16 hr, respectively), mediated by two distinct (but possibly overlapping) cell signaling pathways. Most previous studies focused on the short-term migration involving the G_i_-PI3K pathway, and potentially Ras, Rac1, and other factors [7,9,10,17,27,28,31-35]. LPA-induced cell migration during this time period does not require newly transcribed and/or translated factors. We now demonstrate a distinct signaling pathway leading to LPA-induced long-term cell migration in EOC cells, which was YAP-dependent and relied on transcription and translation to generate factors, including AREG. These new concepts remain to be tested further in additional EOC and other cell types, but are highly significant for both basic science and translational purposes. The EGFR and LPA pathways are likely to overlap and interact in a cell type-dependent manner [35]. We also showed that YAP was required for LPA-induced cell invasion, which is likely to be related to long-term cell migration and requires protease activities.

LPA-induced AREG secretion in a YAP- and time-dependent manner, involved a novel signaling pathway in EOC cells and LPA-EGFR signaling crosstalk. LPA-induced EGFR transactivation was first reported in 1997 [36] and could be mediated through intracellular signals, including Src-mediated signaling [35,37], proteinase-mediated cleavage of pro-EGFR ligands such as pro-heparin-binding EGF-like growth factor (pro-HB-EGF), as well as transcription-dependent processes [38-42]. The proteases (MMP-2, MMP-9, uPA, ADAM1, ADAM 17; expression or shedding) activated by LPA in EOC and other cell types are either up- or down-stream of EGFR [34,35,38,41]. Together with our data, these results suggest that multiple cell type- and time-dependent mechanisms are involved in LPA-EGFR crosstalk. More recently, Rosenbluh *et al.* have shown that β-catenin-driven cancers require a YAP1 transcriptional complex for survival and tumorigenesis [43], further expanding the spectrum of YAP-regulated down-stream targets in colon and other cancers.

Both LPA_1_ and LPA_3_ are involved in LPA-induced YAP activation in HEK293 cells [1]. We showed that LPA_3_, but not LPA_1,_ was needed for LPA-induced YAP activation in OVCA433 EOC cells. This is consistent with the potential negative regulatory role of LPA_1_ in EOC cells revealed by tissue expression and functional assays [7,9,17,44,45]. LPA_3_ has been shown to be coupled predominately to G_q_ proteins and can also be coupled to G_i_ proteins [19-21]. Direct coupling of LPA_3_ to G_12_ and/or G_13_ to activate Rho has not been demonstrated. Our data, however, implies that LPA_3_ may have a direct coupling to G_13_-Rho. While this coupling needs further validation, our data has expanded the understanding of LPA_3_ signaling. The potential roles of LPA_5_ and LPA_6_ in LPA-YAP signaling remain to be tested.

LPA-induced dpYAP could be mediated via inhibition of its kinases (Lats) and/or activation of its protein phosphatase (PP). Phosphorylation of Mst1 (at ser183) or Mst2 (at ser180) is required for their activation; phosphorylation of ser909 of Lats by Mst is required for Lats activity [46]. LPA did not affect Mst activation, consistent with results in HEK293 and MEF cells [1]. While inhibition of Lats1/2 kinase activity was shown to be the major mechanism by which LPA activates YAP in HEK293 cells [1], we found that LPA did not have a significant effect on Lats activation or inhibition [assessed by using the specific phospho-Lats1 (ser909) antibody]. In contrast, our data suggest that activation of PP1A is required for the LPA-YAP effects in EOC cells. Importantly, PPs have *only* been shown to be *negatively* involved in LPA-induced effects previously [47-50]. We presented a *positive* effect of PP in LPA signaling mediated by G_13_ coupling. These apparently trimeric-G protein-dependent and potentially time-dependent negative and positive roles of PPs in LPA signaling are highly intriguing and warrant further studies. In addition, we presented evidence that PP1A is a down-stream target of RhoA-ROCK, either directly or indirectly. It is worth noting that several of the inhibitors/reagents that effectively blocked LPA-induced dpYAP also significantly increased the basal levels of pYAP or total YAP, including Y27632 and C3 (Figure 3), the siRNA against LPA_3_ (Figure 4B), dn-G_13_ (Figure 4C), and OA (Figure 5). These up-regulations were highly reproducible, and suggest that the targeted genes are involved not only in LPA- regulated, but also basal levels of pYAP in these cells. Further understanding of this effect will require additional studies. We showed that LPA was also able to regulate TAZ, a paralog of YAP, in EOC cells, although the current work is mainly focused on YAP. Interestingly, while the LPA-dpYAP effect peaked at 1–2 hr in EOC cells, the dpTAZ effect induced by LPA in OVCA433 persisted at 4 hrs, suggesting that these two proteins may have overlapping and distinct cellular effects. The role of TAZ in EOC remains to be studied.

LPA is involved in many aspects of EOC pathogenesis and development and is a major target for EOC treatment [14,51,52]. Further study of the signaling mechanisms of LPA will be important for basic science as well as for translational applications. Although YAP has been implicated as an oncogene in EOC [5,6], its regulation in EOC cells was totally unknown. While LPA is a confirmed target in EOC [51,53], how to target LPA in EOC has been under hot pursuit. In particular, since LPA is small molecular lipid and has a very broad spectrum of normal physiological roles, targeting LPA effectively and selectively for cancer treatment is a great challenge. If the YAP pathway is further confirmed to mediate the important roles of LPA in EOC, alternative approaches can be developed.

The majority of EOC subtypes examined here were serous (24 of 27), with one endometriod, one stromal and one unknown (Table 2). Zhang *et al.* have shown that the association of YAP with poor survival is predominantly in clear cell tumors, independent of stage [6]. We did not have the clear cell subtype in our study, but we observed a definite decrease in pYAP in EOC serous tumors as compared to control tissues, suggesting that pYAP might be also a good marker for identifying the predominant and deadly serous EOC. In particular, one of the challenges in EOC diagnosis and monitoring is the difficulty of distinguishing benign from malignant ovarian or other gynecological (GYN) diseases. Our results shown in Figure 7, although limited in the number of samples, suggest that reduce dpYAP is specifically associated with malignancy with less or no involvement in benign GYN diseases. The data remain to be validated in larger cohorts.

## Conclusions

Although LPA has been shown to be an extracellular regulator of the Hippo-YAP pathways in HEK293 and breast cancer cells [1], the current studies not only represent the first demonstration of LPA-YAP signaling in EOC cells, but also reveal several innovative aspects of this signaling. These include the new concept of short- versus long-term cell migration induced by LPA; LPA_3_-G_13_ coupled signaling; RhoA-ROCK as an upstream regulator of PP1A; PP1A, but not Lats kinase, as a major regulator for LPA-induced dpYAP; and AREG as a down-stream YAP effector to mediate LPA-induced long-term cell migration of EOC cells. In addition, we tested and confirmed dpYAP expression in human EOC [5,6], further demonstrating the translational potential of this pathway.

## Methods

### Human tissues

Normal ovary, benign ovary, and ovarian cancer tissues were purchased from the Cooperative Human Tissue Network (CHTN; Philadelphia, PA); the usage of these tissues was approved by an Indiana University School of Medicine IRB. All tissues were pathologically examined. Among the 10 benign tissue samples used, three were cystadenoma, three were fibroma, and others were endometriosis, cortical inclusion cysts, Brenner tumor and cystadenofibroma.

### Reagents

Oleoyl-LPA was purchased from Avanti Polar Lipids (Birmingham, AL). The following inhibitors or reagents were used in this study: SB203580, PD98059, and LY294002 (Enzo Life Sciences, Farmingdale, NY), MK2206 and Y27632 (Biovision, Milpitas, CA), C3 exoenzyme (Cytoskeleton, Denver, CO), Ki16425 and PD153035 (Selleckchem, Houston, TX), okadaic acid (OA) and AG1478 (Calbiochem, San Diego, CA), pertussis toxin (PTX; Invitrogen, Grand Island, NY), actinomycin D (ActD) and cyclohexamide (CHX; Sigma-Aldrich, St. Louis, MO). YAP, phospho-YAP (Ser127), phospho-Lats1 (Ser909), phospho-Mst1 (Thr183), phospho-Mst2 (Thr180), RhoA(67B9), and PP2A antibodies were from Cell Signaling (Boston, MA). GAPDH, p-TAZ (Ser89) and PP1 antibodies were from Santa Cruz Biotechnology (Santa Cruz, CA). Alexa fluor secondary antibodies were from Life Technologies, Grand Island, NY. The dn and ca forms of large and small G protein constructs were from UMR cDNA Resource Center (Rolla, MO).

### Cell lines and culture

The OVCA433 cells were obtained from Dr. R. Bast (M.D. Anderson) and the OVCAR5 cells were obtained from ATCC (Manassas, VA). Both cells lines were tested and authenticated in 2012 by Biosynthesis, Inc. (Lewisville, TX) using short tandem repeat analysis. Monty-1 is a primary EOC cell line developed and given to us by Dr. E. Lengyel (University of Chicago) [54]. All cell lines were maintained in a humidified atmosphere at 37°C with 5% CO_2_. OVCA433 cells were cultured in RPMI 1640 with glutamine, 10% FBS (ATCC, Manassas, VA), and 100 μg/mL penicillin/streptomycin (P/S). CAOV3, OVCAR5 and Monty-1 cells were cultured in DMEM with glutamine, 10% FBS (ATCC, Manassas, VA), and 100 μg/mL P/S. For serum starvation, cells were incubated in growth medium without FBS or antibiotics. LPA treatment was always in cells starved from serum for 16 hr.

### Western blot analyses

Western blot analyses were conducted using standard procedures and proteins were detected using primary antibodies and fluorescent secondary antibodies (IRDye800CW-conjugated or IRDye680-conjugated anti-species IgG, Li-Cor Biosciences, Lincoln, NE). The fluorescent signals were captured on an Odyssey Infrared Imaging System (Li-Cor Biosciences, Lincoln, NE) with both 700- and 800-nm channels. Boxes were manually placed around each band of interest, and the software returned near-infrared fluorescent values of raw intensity with background subtraction (Odyssey 3.0 analytical software, Li-Cor Biosciences, Lincoln, NE).

### Immunofluorescence staining

OVCAR433 or OVCAR5 cells were seeded in chamber slides. After treatment, cells were fixed with 4% paraformaldehyde-PBS for 15 min. Following blocking in 5% goat serum with 0.3% Triton X-100 in PBS for 60 min, cells were incubated with YAP primary antibody (1:100 dilution) overnight at 4°C. After three washes with PBS, cells were incubated with Alexa Fluor 488- or 555-conjugated secondary antibodies (Invitrogen, 1:500 dilution) for 2 hr at room temperature. Slides were then washed three times and mounted. Immunofluorescence was detected using a Qimage Retiga 2000Rcamera (Surrey, BC, Canada) at 60× magnification). For frozen tissues, 5 μm sections were prepared and subjected to immunostaining as described.

### Immunohistochemistry (IHC) analysis

The formalin-fixed paraffin-embedded sections (5 μm thick) of the normal ovaries, ovaries with benign diseases, or ovarian cancer were analyzed by IHC using the primary YAP or p-YAP antibody (1:100) and a biotin-conjugated secondary antibody. For IHC quantification, the sections were analyzed using Nikon TE2000-s microscope (Melville, NY). Four randomly selected areas were photographed at 40× magnification using a Qimage Retiga 2000Rcamera (Surrey, BC, Canada). The images were analyzed using the Image-Pro Plus image analysis software (Media Cybernetics, Rockville, MD).

### DNA and RNA transfection

6-well plates were seeded with 5 × 10^4^cell/well in 2 mL media 24 hr before transfection; cells were 80%–90% confluent. Cells were transfected with siRNA (100 pmol/well) or plasmid DNA (4 μg/well) using Lipofectamine 2000 Reagent (Life Technologies, Grand Island, NY) according to manufacturer’s instruction. After 48 hr of transfection, cells were starved for migration and invasion assays. All siRNAs were purchased from Santa Cruz Biotechnology (Santa Cruz, CA).

### Cell migration and invasion assays

Migration and invasion assays were conducted using Transwell plates with 8 μm pore size membranes (Corning Inc., Corning, NY) as described previously [10]. After incubation for 4 or 16 hr (for migration assays) or 24 hr (for invasion assays), cells remaining in the upper side of the filter were removed with cotton swabs. The cells attached on the lower surface were fixed and stained using crystal violet and washed with water. Cells were counted with five high power fields per membrane and results were presented as the mean number of cells migrated per field per membrane. All experiments were conducted in triplicate.

### Quantitative real-time PCR

After siRNA transfection for 48 hr, cells were washed with cold PBS and collected in the Qiagen RLT lysis buffer (Qiagen, Valencia, CA). RNA was extracted with an RNeasy mini kit (Qiagen, Valencia, CA) and reverse transcribed by M-MLV reverse transcriptase. Quantitative real-time PCR was performed on a Light Cycler 480 (Roche, Indianapolis, IN) with a SYBR Green I Master Mix (Roche, Indianapolis, IN). mRNA abundance was normalized to GAPDH. Negative controls contained no transcript or reverse transcriptase. RNA from three separate cell pellets per treatment was analyzed. Relative gene expression was calculated using the method given in Applied Biosystems User Bulletin No. 2. (P/N 4303859B), with non-targeting siRNA-treated cells acting as the control in each data set. Primer pairs used in this study were: GAPDH: F, 5^′^-GAAGGTGAAGGTCGGAGT-3^′^/R, 5^′^-GAAGATGGTGATGGGATTTC-3^′^; LPA_1_: F, 5^′^-AATCGAGAGGCACATTACGG-3^′^/R, 5^′^-GTTGAAAATGGCCCAGAAGA-3^′^; LPA_2_: F, 5^′^-TTGTCTTCCTGCTCATGGTG-3^′^/R, 5^′^-TCAGCATCTCGGCAAGAGTA-3^′^; LPA_3_: F, 5^′^-TGCTCATTTTGCTTGTCTGG-3^′^/R, 5^′^-GCCATACATGTCCTCGTCCT-3^′^; LPA_4_: F, 5^′^-CTTCGCAAGCCTGCTACTCT-3^′^/R, 5^′^-GGCTTTGTGGTCAAAGGTGT-3^′^; AREG: F, 5^′^-GGGAGTGAGATTTCCCCTGT-3^′^/R, 5^′^-AGCCAGGTATTTGTGGTTCG-3^′^.

### AREG ELISA

Conditioned media were collected and stored at −80°C until ELISA assays were conducted. ELISA assays were performed using a Human Amphiregulin DuoSet ELISA Development System (R&D Systems. Minneapolis, MN) in triplicate wells according to the manufacturer’s instructions. The optical density at 450 nm was measured on an automated plate reader (PerkinElmer, Santa Clara, CA). Experiments were repeated three times.

### Statistical analyses

The Student’s *t*-test was utilized to assess the statistical significance of the difference between two treatments. A *P* value of less than 0.05 was considered significant.

## Abbreviations

AREG: Amphiregulin; dpYAP: YAP dephosphorylation; dpTAZ: TAZ dephosphorylation; EGFR: Epidermal growth factor receptor; EOC: Epithelial ovarian cancer; LPA: Lysophosphaditic acid; ROCK: Rho-associated protein kinase; PP: Protein phosphatase; YAP: Yes-associated protein.

## Competing interests

The authors declare no completing interests related to this work.

## Authors’ contributions

HC conducted all the experiments presented in this work. YX made central contributions to conception and design of the studies; analysis and interpretation of data. Both authors were involved in drafting the manuscript and giving final approval of the version to be published. Each author has participated sufficiently in the work to take public responsibility for appropriate portions of the content.

## References

[B1] Yu FX Zhao B Panupinthu N Jewell JL Lian I Wang LH Zhao J Yuan H Tumaneng K Li H Regulation of the Hippo-YAP Pathway by G-Protein-Coupled Receptor Signaling Cell 2012 150 780 791 10.1016/j.cell.2012.06.037 22863277 PMC3433174

[B2] Sudol M Yes-associated protein (YAP65) is a proline-rich phosphoprotein that binds to the SH3 domain of the Yes proto-oncogene product Oncogene 1994 9 2145 2152 8035999

[B3] Wang K Degerny C Xu M Yang XJ YAP, TAZ, and Yorkie: a conserved family of signal-responsive transcriptional coregulators in animal development and human disease Biochem Cell Biol 2009 87 77 91 10.1139/O08-114 19234525

[B4] Miller E Yang J Deran M Wu C Su AI Bonamy GM Liu J Peters EC Wu X Identification of Serum-Derived Sphingosine-1-Phosphate as a Small Molecule Regulator of YAP Chem Biol 2012 19 955 962 10.1016/j.chembiol.2012.07.005 22884261

[B5] Hall CA Wang R Miao J Oliva E Shen X Wheeler T Hilsenbeck SG Orsulic S Goode S Hippo pathway effector Yap is an ovarian cancer oncogene Cancer Res 2010 70 8517 8525 10.1158/0008-5472.CAN-10-1242 20947521 PMC2970655

[B6] Zhang X George J Deb S Degoutin JL Takano EA Fox SB Bowtell DD Harvey KF The Hippo pathway transcriptional co-activator, YAP, is an ovarian cancer oncogene Oncogene 2011 30 2810 2822 10.1038/onc.2011.8 21317925

[B7] Kim KS Sengupta S Berk M Kwak YG Escobar PF Belinson J Mok SC Xu Y Hypoxia enhances lysophosphatidic acid responsiveness in ovarian cancer cells and lysophosphatidic acid induces ovarian tumor metastasis in vivo Cancer Res 2006 66 7983 7990 10.1158/0008-5472.CAN-05-4381 16912173

[B8] Li H Zhao Z Wei G Yan L Wang D Zhang H Sandusky GE Turk J Xu Y Group VIA phospholipase A2 in both host and tumor cells is involved in ovarian cancer development FASEB J 2010 24 4103 4116 10.1096/fj.10-161356 20530749 PMC2996900

[B9] Ren J Xiao YJ Singh LS Zhao X Zhao Z Feng L Rose TM Prestwich GD Xu Y Lysophosphatidic acid is constitutively produced by human peritoneal mesothelial cells and enhances adhesion, migration, and invasion of ovarian cancer cells Cancer Res 2006 66 3006 3014 10.1158/0008-5472.CAN-05-1292 16540649

[B10] Sengupta S Xiao YJ Xu Y A novel laminin-induced LPA autocrine loop in the migration of ovarian cancer cells FASEB J 2003 17 1570 1572 12824286 10.1096/fj.02-1145fje

[B11] Xu X Prestwich GD Inhibition of tumor growth and angiogenesis by a lysophosphatidic acid antagonist in an engineered three-dimensional lung cancer xenograft model Cancer 2010 116 1739 1750 10.1002/cncr.24907 20143443 PMC2847044

[B12] Xu Y Gaudette DC Boynton JD Frankel A Fang XJ Sharma A Hurteau J Casey G Goodbody A Mellors A Characterization of an ovarian cancer activating factor in ascites from ovarian cancer patients Clin Cancer Res 1995 1 1223 1232 9815916

[B13] Xu Y Shen Z Wiper DW Wu M Morton RE Elson P Kennedy AW Belinson J Markman M Casey G Lysophosphatidic acid as a potential biomarker for ovarian and other gynecologic cancers JAMA 1998 280 719 723 10.1001/jama.280.8.719 9728644

[B14] Xu Y Xiao YJ Zhu K Baudhuin LM Lu J Hong G Kim KS Cristina KL Song L Williams FS Unfolding the pathophysiological role of bioactive lysophospholipids Curr Drug Targets Immune Endocr Metabol Disord 2003 3 23 32 10.2174/1568008033340414 12570723

[B15] Ishii S Noguchi K Yanagida K Non-Edg family lysophosphatidic acid (LPA) receptors Prostaglandins Other Lipid Mediat 2009 89 57 65 10.1016/j.prostaglandins.2009.06.001 19524700

[B16] Yanagida K Kurikawa Y Shimizu T Ishii S Current progress in non-Edg family LPA receptor research Biochim Biophys Acta 2013 1831 33 41 10.1016/j.bbalip.2012.08.003 22902318

[B17] Sengupta S Kim KS Berk MP Oates R Escobar P Belinson J Li W Lindner DJ Williams B Xu Y Lysophosphatidic acid downregulates tissue inhibitor of metalloproteinases, which are negatively involved in lysophosphatidic acid-induced cell invasion Oncogene 2007 26 2894 2901 10.1038/sj.onc.1210093 17130843

[B18] Cai Q Zhao Z Antalis C Yan L Del Priore G Hamed AH Stehman FB Schilder JM Xu Y Elevated and secreted phospholipase A(2) activities as new potential therapeutic targets in human epithelial ovarian cancer FASEB J 2012 26 3306 3320 10.1096/fj.12-207597 22767227 PMC3405265

[B19] Choi JW Chun J Lysophospholipids and their receptors in the central nervous system Biochim Biophys Acta 2013 1831 20 32 10.1016/j.bbalip.2012.07.015 22884303 PMC3693945

[B20] Bandoh K Aoki J Hosono H Kobayashi S Kobayashi T Murakami-Murofushi K Tsujimoto M Arai H Inoue K Molecular cloning and characterization of a novel human G-protein-coupled receptor, EDG7, for lysophosphatidic acid J Biol Chem 1999 274 27776 27785 10.1074/jbc.274.39.27776 10488122

[B21] Im DS Heise CE Harding MA George SR O’Dowd BF Theodorescu D Lynch KR Molecular cloning and characterization of a lysophosphatidic acid receptor, Edg-7, expressed in prostate Mol Pharmacol 2000 57 753 759 10727522

[B22] Yang N Morrison CD Liu P Miecznikowski J Bshara W Han S Zhu Q Omilian AR Li X Zhang J TAZ induces growth factor-independent proliferation through activation of EGFR ligand amphiregulin Cell Cycle 2012 11 2922 2930 10.4161/cc.21386 22825057 PMC3419062

[B23] Zhang J Ji JY Yu M Overholtzer M Smolen GA Wang R Brugge JS Dyson NJ Haber DA YAP-dependent induction of amphiregulin identifies a non-cell-autonomous component of the Hippo pathway Nat Cell Biol 2009 11 1444 1450 10.1038/ncb1993 19935651 PMC2819909

[B24] Noguchi K Herr D Mutoh T Chun J Lysophosphatidic acid (LPA) and its receptors Curr Opin Pharmacol 2009 9 15 23 10.1016/j.coph.2008.11.010 19119080

[B25] Wang P Bai Y Song B Wang Y Liu D Lai Y Bi X Yuan Z PP1A-mediated dephosphorylation positively regulates YAP2 activity PLoS One 2011 6 e24288 10.1371/journal.pone.0024288 21909427 PMC3164728

[B26] Stoll SW Benedict M Mitra R Hiniker A Elder JT Nunez G EGF receptor signaling inhibits keratinocyte apoptosis: evidence for mediation by Bcl-XL Oncogene 1998 16 1493 1499 10.1038/sj.onc.1201657 9580112

[B27] Sengupta S Wang Z Tipps R Xu Y Biology of LPA in health and disease Semin Cell Dev Biol 2004 15 503 512 10.1016/j.semcdb.2004.05.003 15271295

[B28] Zhao X Wang D Zhao Z Xiao Y Sengupta S Xiao Y Zhang R Lauber K Wesselborg S Feng L Caspase-3-dependent activation of calcium-independent phospholipase A2 enhances cell migration in non-apoptotic ovarian cancer cells J Biol Chem 2006 281 29357 29368 10.1074/jbc.M513105200 16882668

[B29] Stahle M Veit C Bachfischer U Schierling K Skripczynski B Hall A Gierschik P Giehl K Mechanisms in LPA-induced tumor cell migration: critical role of phosphorylated ERK J Cell Sci 2003 116 3835 3846 10.1242/jcs.00679 12902401

[B30] Van Leeuwen FN Giepmans BN Van Meeteren LA Moolenaar WH Lysophosphatidic acid: mitogen and motility factor Biochem Soc Trans 2003 31 1209 1212 10.1042/BST0311209 14641027

[B31] Bian D Su S Mahanivong C Cheng RK Han Q Pan ZK Sun P Huang S Lysophosphatidic Acid Stimulates Ovarian Cancer Cell Migration via a Ras-MEK Kinase 1 Pathway Cancer Res 2004 64 4209 4217 10.1158/0008-5472.CAN-04-0060 15205333

[B32] So J Navari J Wang FQ Fishman DA Lysophosphatidic acid enhances epithelial ovarian carcinoma invasion through the increased expression of interleukin-8 Gynecol Oncol 2004 95 314 322 10.1016/j.ygyno.2004.08.001 15491751

[B33] So J Wang FQ Navari J Schreher J Fishman DA LPA-induced epithelial ovarian cancer (EOC) in vitro invasion and migration are mediated by VEGF receptor-2 (VEGF-R2) Gynecol Oncol 2005 97 870 878 10.1016/j.ygyno.2005.03.004 15919106

[B34] Do TV Symowicz JC Berman DM Liotta LA Petricoin EF 3rd Stack MS Fishman DA Lysophosphatidic acid down-regulates stress fibers and up-regulates pro-matrix metalloproteinase-2 activation in ovarian cancer cells Mol Cancer Res 2007 5 121 131 10.1158/1541-7786.MCR-06-0319 17314270

[B35] Jeong KJ Cho KH Panupinthu N Kim H Kang J Park CG Mills GB Lee HY EGFR mediates LPA-induced proteolytic enzyme expression and ovarian cancer invasion: Inhibition by resveratrol Mol Oncol 2013 7 121 129 10.1016/j.molonc.2012.10.001 23127547 PMC5528397

[B36] Daub H Wallasch C Lankenau A Herrlich A Ullrich A Signal characteristics of G protein-transactivated EGF receptor EMBO J 1997 16 7032 7044 10.1093/emboj/16.23.7032 9384582 PMC1170306

[B37] Luttrell LM Daaka Y Lefkowitz RJ Regulation of tyrosine kinase cascades by G-protein-coupled receptors Curr Opin Cell Biol 1999 11 177 183 10.1016/S0955-0674(99)80023-4 10209148

[B38] Prenzel N Zwick E Daub H Leserer M Abraham R Wallasch C Ullrich A EGF receptor transactivation by G-protein-coupled receptors requires metalloproteinase cleavage of proHB-EGF Nature 1999 402 884 888 10622253 10.1038/47260

[B39] Inoue A Ishiguro J Kitamura H Arima N Okutani M Shuto A Higashiyama S Ohwada T Arai H Makide K Aoki J TGFalpha shedding assay: an accurate and versatile method for detecting GPCR activation Nat Methods 2012 9 1021 1029 10.1038/nmeth.2172 22983457

[B40] Shiomi T Boudreault F Padem N Higashiyama S Drazen JM Tschumperlin DJ Lysophosphatidic acid stimulates epidermal growth factor-family ectodomain shedding and paracrine signaling from human lung fibroblasts Wound Repair Regen 2011 19 229 240 10.1111/j.1524-475X.2010.00655.x 21362091 PMC3076689

[B41] Yin J Yu FS ERK1/2 mediate wounding- and G-protein-coupled receptor ligands-induced EGFR activation via regulating ADAM17 and HB-EGF shedding Invest Ophthalmol Vis Sci 2009 50 132 139 18658095 10.1167/iovs.08-2246 PMC3656386

[B42] Ediger TL Danforth BL Toews ML Lysophosphatidic acid upregulates the epidermal growth factor receptor in human airway smooth muscle cells Am J Physiol Lung Cell Mol Physiol 2002 282 L91 98 11741820 10.1152/ajplung.2002.282.1.L91

[B43] Rosenbluh J Nijhawan D Cox AG Li X Neal JT Schafer EJ Zack TI Wang X Tsherniak A Schinzel AC beta-Catenin-driven cancers require a YAP1 transcriptional complex for survival and tumorigenesis Cell 2012 151 1457 1473 10.1016/j.cell.2012.11.026 23245941 PMC3530160

[B44] Mills GB Eder A Fang X Hasegawa Y Mao M Lu Y Tanyi J Tabassam FH Wiener J Lapushin R Critical role of lysophospholipids in the pathophysiology, diagnosis, and management of ovarian cancer Cancer Treat Res 2002 107 259 283 11775454 10.1007/978-1-4757-3587-1_12

[B45] Fang X Gaudette D Furui T Mao M Estrella V Eder A Pustilnik T Sasagawa T Lapushin R Yu S Lysophospholipid growth factors in the initiation, progression, metastases, and management of ovarian cancer Ann N Y Acad Sci 2000 905 188 208 10818454 10.1111/j.1749-6632.2000.tb06550.x

[B46] Avruch J Zhou D Fitamant J Bardeesy N Mou F Barrufet LR Protein kinases of the Hippo pathway: regulation and substrates Semin Cell Dev Biol 2012 23 770 784 10.1016/j.semcdb.2012.07.002 22898666 PMC3489012

[B47] Hahnenkamp K Durieux ME Van Aken H Berning S Heyse TJ Honemann CW Linck B Modulation of Xenopus laevis Ca-activated Cl currents by protein kinase C and protein phosphatases: implications for studies of anesthetic mechanisms Anesth Analg 2004 99 416 422 table of contents 15271716 10.1213/01.ANE.0000121351.38401.AB

[B48] Park SJ Itoh T Takenawa T Phosphatidylinositol 4-phosphate 5-kinase type I is regulated through phosphorylation response by extracellular stimuli J Biol Chem 2001 276 4781 4787 10.1074/jbc.M010177200 11087761

[B49] Mattingly RR Saini V Macara IG Activation of the Ras-GRF/CDC25Mm exchange factor by lysophosphatidic acid Cell Signal 1999 11 603 610 10.1016/S0898-6568(99)00034-0 10433521

[B50] Fleming IN Elliott CM Buchanan FG Downes CP Exton JH Ca2+/calmodulin-dependent protein kinase II regulates Tiam1 by reversible protein phosphorylation J Biol Chem 1999 274 12753 12758 10.1074/jbc.274.18.12753 10212259

[B51] Mills GB Moolenaar WH The emerging role of lysophosphatidic acid in cancer Nat Rev Cancer 2003 3 582 591 12894246 10.1038/nrc1143

[B52] Xu Y Wang D Wang Z Stack MS, Fishman DA Lipid generation and signaling in ovarian cacner Cancer Treatment and Research-Ovarian cancer 2009 2 New York, Dordrecht, Heidelberg, London: Springer 241 268 10.1007/978-0-387-98094-2_12 19763440

[B53] Fang X Schummer M Mao M Yu S Tabassam FH Swaby R Hasegawa Y Tanyi JL LaPushin R Eder A Lysophosphatidic acid is a bioactive mediator in ovarian cancer Biochim Biophys Acta 2002 1582 257 264 10.1016/S1388-1981(02)00179-8 12069836

[B54] Kaur S Kenny HA Jagadeeswaran S Zillhardt MR Montag AG Kistner E Yamada SD Mitra AK Lengyel E {beta}3-integrin expression on tumor cells inhibits tumor progression, reduces metastasis, and is associated with a favorable prognosis in patients with ovarian cancer Am J Pathol 2009 175 2184 2196 10.2353/ajpath.2009.090028 19808644 PMC2774080

