# Correction: The role of LPA and YAP signaling in long-term migration of human ovarian cancer cells

**DOI:** 10.1186/1478-811X-11-92

**Published:** 2013-12-13

**Authors:** Hui Cai, Yan Xu

**Affiliations:** 1 First Affiliated Hospital, Xi’an Jiaotong University, Xi’an, China; 2 Department of Obstetrics and Gynecology, Indiana University School of Medicine, 975 W. Walnut St. IB355A, Indianapolis, IN 46202, USA

## Correction

In the original paper published [1], there is a mistake in Figure 4. Figure 4D and Figure 4E are the same, but Figure 4E should have been different (the figures show two different cell lines). Figure 1 in this correction article is the correct version of Figure 4 from the original article [1]. The figure legend does not need to be changed.

**Figure 1:**
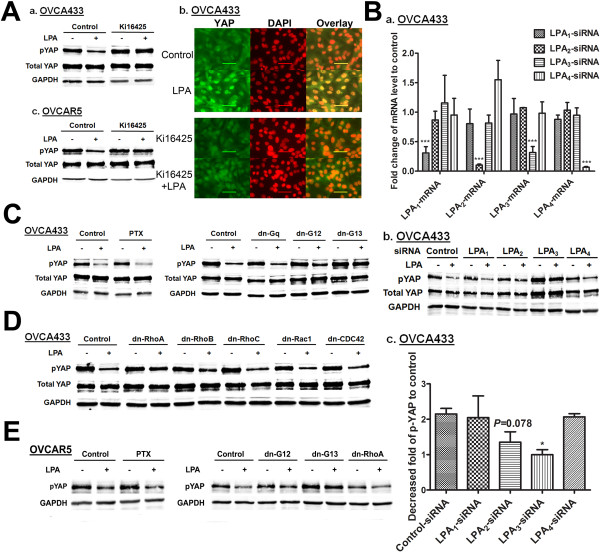
**LPA**_**3**_**, but not or to lesser extent LPA**_**1**_**, LPA**_**2**_**, and LPA**_**4**_**, mediated the LPA-dpYAP effect. A,** OVCA433 **(a)** and OVCAR5 **(c)** cells were starved and pretreated with Ki16425 (10 μM) for 1 hr prior to treatment with LPA (10 μM, 2 hr). pYAP was analyzed by Western blot. **(b)** The effect of Ki16425 on LPA (10 μM, 2 hr)-induced YAP nuclear translocation in OVCA433 cells. Green: YAP; red: DAPI. Representative results are shown. **B, (a)** The mRNA levels of LPA receptors after siRNA-treatment in OVCAR433 cells were determined by quantitative real-time PCR. Normalized expression values are given as percentage of control siRNA treated samples (means ± SD of three independent experiments). ***P < 0.001. **(b)** LPA (10 μM, 2 hr)-induced dpYAP effects were determined in LPA receptor specific siRNA-treated cells (48 hr post-transfection). **(c)** Quantitation of Western blots from **(b)** presented as fold decrease of pYAP after LPA stimulation compared to unstimulated controls. The data are means ± SD from three independent experiments. *P < 0.05. **C, D** and **E,** Cells were pretreated with PTX (100 ng/mL, 16 hr) or transfected with different dn plasmids for 48 hr, starved and then treated with LPA (10 μM, 2 hr). Cell lysates were analyzed by Western blot. Representative results are shown.
